# Ectopic follicular variant of papillary thyroid carcinoma in anterior mediastinum with a normal thyroid gland. A case report

**DOI:** 10.1016/j.ijscr.2018.08.051

**Published:** 2018-08-30

**Authors:** Farida Karim, Hina Inam, Usama Khalid Choudry, Saulat Hasnain Fatimi

**Affiliations:** a Department of Opthalmology, Aga Khan University Hospital, Pakistan; b Department of General Surgery, Shifa International Hospital, Pakistan; c Department of Cardiothoracic surgery, Aga Khan University Hospital, Pakistan

**Keywords:** Ectopic papillary thyroid carcinoma, Mediastinal tumor, Normal thyroid gland

## Abstract

**Introduction:**

Ectopic thyroid is rare, especially in the anterior mediastinum. Furthermore, true malignant transformation in ectopic location with normal orthotopic gland is exceptionally unusual. The authors have tried to put forward a challenging case of a malignant mediastinal mass managed successfully via a multidisciplinary approach.

**Presentation of case:**

We report the case of a 55 year old female with ectopic follicular variant of papillary thyroid cancer in the anterior mediastinum with no disease in the thyroid gland. Subsequently, complete excision of the mediastinal mass and total thyroidectomy was done followed by radio-iodine ablation with radiotherapy. Patient showed good uneventful recovery and remains tumor free till date.

**Conclusion:**

The aim of this report is to bring forward malignant ectopic thyroid carcinoma as a differential for malignant mediastinal masses. It is emphasized that the diagnosis is made essentially through physical and pathological examination and, in most cases, only after surgery. A Multidisciplinary approach is recommended in such cases.

## Highlights

• Ectopic papillary thyroid carcinoma is a rare finding in the mediastinum.
• There is a possibility of a normal orthotopic thyroid gland with ectopic thyroid cancer.
• Complete tumor excision followed by radio ablative iodine provides satisfactory results.

## 1 Introduction

The following case report has been reported from our University Hospital, which is an internationally recognized teaching hospital and a tertiary care center based in Pakistan, in accordance with the SCARE guidelines for case reports [15]. Ectopic thyroid tissue is a rare entity resulting from developmental defects at early stages of thyroid gland embryogenesis, during its passage from the floor of the primitive foregut to its final pre-tracheal position. It is frequently found around the course of the thyroglossal duct or laterally in the neck, as well as in distant places such as the mediastinum and the subdiaphragmatic organs [1]. However, true malignant transformation in ectopic thyroid tissue is extremely rare [2]. Primary thyroid carcinomas arising from ectopic thyroid tissue are uncommon and have been reported to arise from thyroid tissue in the thyroglossal cysts, lateral aberrant thyroid tissue, lingual thyroid, mediastinum and struma ovarii. Most tumors in the ectopic locations include papillary carcinomas, mixed follicular or Hurthle cell tumors [3].

One challenge associated with such infrequent manifestation is the choice of appropriate diagnostic approach. Radiographic modalities are better suited for such infrequent diagnoses. Scintigraphy, using Tc-99m, I-131, or I-123, is the most important diagnostic tool to detect ectopic thyroid tissue and shows the absence or presence of thyroid in its normal location. Thyroid scan can also unmask additional sites of thyroid tissue [4]. CT and MRI are valuable tools in identifying the site of ectopy, especially when it is distant from the descending pathway of thyroid [1]. Fine needle aspiration cytology (FNAC) provides considerable assistance in confirming the diagnosis of ectopic thyroid. It is the only modality to differentiate between a benign and a malignant lesion [5]. The most useful immunohistological marker is anti-thyroglobulin (Tg) [1].

The differentiation between carcinoma arising in ectopic thyroid tissue and a metastatic carcinoma is difficult. The diagnosis can be made indirectly by taking some features into account, such as separate blood supply of the ectopic gland from extra-cervical vessels, no personal history of malignancy, and normal or absent orthotopic thyroid with no history of surgery [6].

Owing to the rarity of this clinical condition, no unanimity about the optimal therapeutic strategy exists. Intrathoracic goiter is managed surgically. Its removal usually necessitates thoracotomy or sternotomy [7] Mediastinal ectopic seems to be curable if complete resection is performed [8]. For residual or metastatic/recurrent disease, radioiodine therapy may have favorable outcome. In more refractory cases, such as those with multiple metastatic lesions or those which absorb radioiodine poorly, external beam radiation is a reasonable approach [9].

## 2 Presentation of case

**Fig. 1:**
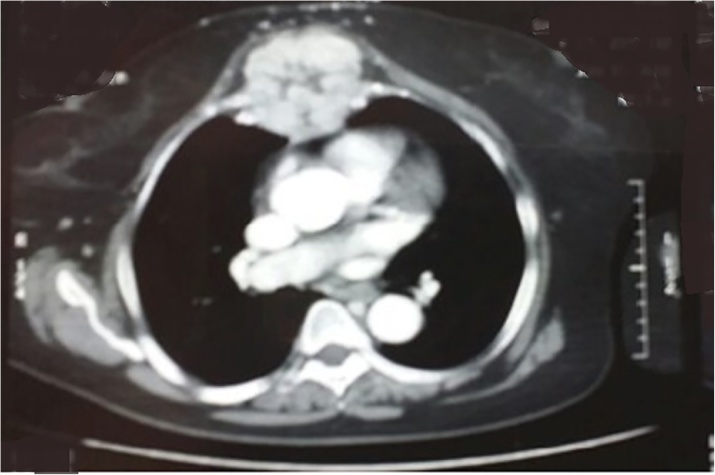
CT scan of chest showing a mediastinal mass.

**Fig. 2:**
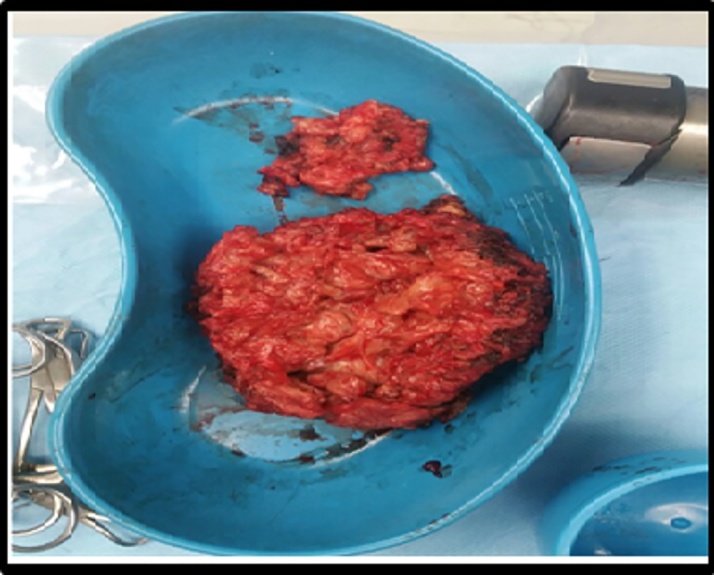
Gross morphology showing a highly vascular soft mass.

**Fig. 3:**
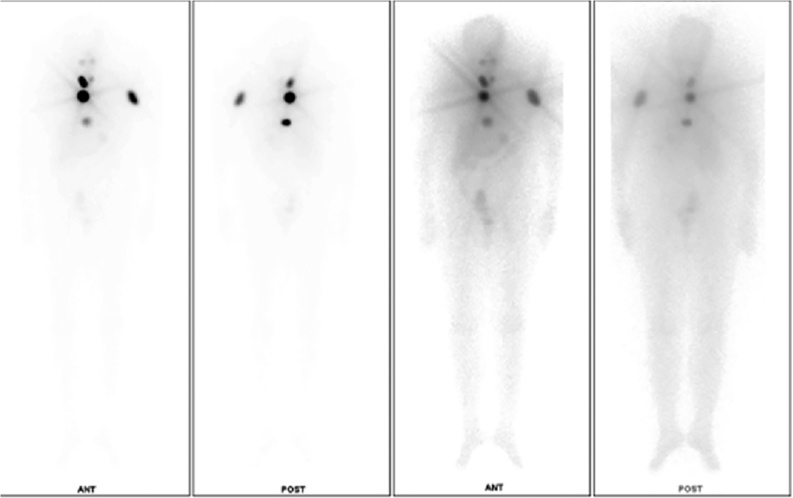
Post radiotherapy bone scan showing good iodine uptake.

**Fig. 4:**
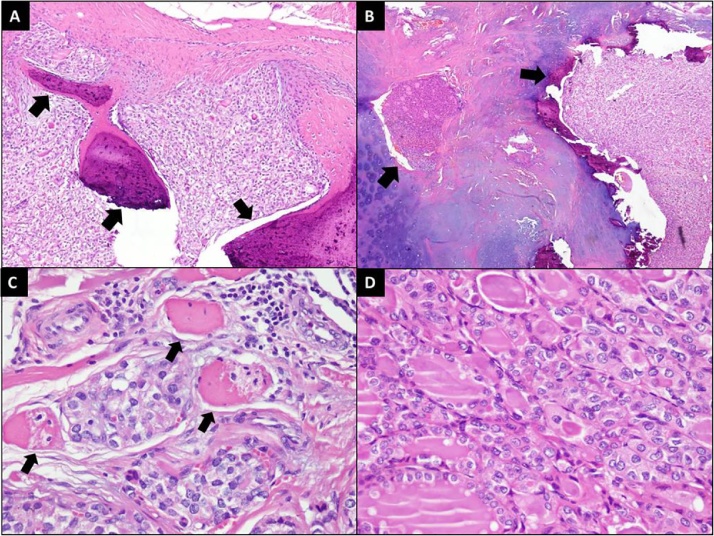
**[A]** Tumor infiltration into bone (arrows). (HnE stain;100× magnification) **[B]** Tumor infiltration into cartilage (arrows). (HnE stain;40× magnification) **[C]** Tumor infiltration into muscle fibers (arrows). (HnE stain; 400× magnification) **[D]** Tumor cells showing follicular arrangement and nuclei exhibiting enlargement, overlapping and clearing. (HnE stain;400× magnification).

A 55-year old female (BMI: 30.7 kg/m^2^) presented with a progressively increasing mediastinal mass for the last two years. On examination, the mass was mobile, non-tender, fixed on center portion of sternal body, well circumscribed and firm, measuring 15*5 cm, protruding out of the anterior chest wall. Her history was significant for a right thyroid lobectomy for benign goiter. The rest of past medical history was unremarkable and she did not show any signs/symptoms of hypo or hyperthyroidism (Fig. 1, Fig. 2, Fig. 3, Fig. 4) .

A CT scan of chest, abdomen and pelvis showed a 15*15 cm localized anterior mediastinal mass invading through the sternal bone in the center and protruding outward from the skin (Fig. 1). On CT Scan the thyroid appeared benign.

A trucut biopsy of the anterior mediastinal mass showed cores of thyroid tissue with TTF-1 positive cells – a characteristic of thyroid follicular cells. Her initial thyroid profile was within normal limits, however, she had raised thyroglobulin antibodies (>6000 ng/ml). Patient also had a bone scan which showed metastasis in ribs and long bone.

At this time the case was discussed in tumor board meeting and it was mutually decided that this patient will benefit from a complete excision of the mediastinal mass, total thyroidectomy followed by radio-iodine ablation with radiotherapy which will take care of the residual metastatic disease in bone. Patient underwent a mid-sternal body excision along with complete removal of tumor and total thyroidectomy. The sternum was reconstructed with prolene mesh and sternal wires and the skin was closed primarily. Intraoperatively, a soft mass was identified in front of the sternum and anterior mediastinum which was highly vascular with feeding vessels coming from bilateral internal mammary arteries, without any connection to the thyroid gland. The surgery lasted 4 h, patient developed bleeding from both internal thoracic arteries, which was controlled eventually, surgery was technically challenging owing to the close proximity to mediastinal structures with the tumor. Post operatively patient complained of pain and developed bilateral atelectasis secondary to paradoxical breathing. The specimen was sent for biopsy (See Fig. 2). Postoperatively, she had an uneventful course. The biopsy of the thyroid specimen showed benign thyroid tissue, however, the mediastinal mass showed follicular variant of papillary thyroid cancer (See Fig. 4).

Radioablation was done as per the plan after 6 weeks of surgery. A dosage of 80 mCi was administered to the patient. With a post therapy scan showing good uptake of therapeutic dose by post-operative residual functioning thyroid tissue over thyroid bed (See Fig. 3). Patient continues to do well without any revival of disease after 9 months.

## 3 Discussion

Our patient presented with an asymptomatic mediastinal mass making the diagnosis extremely challenging. Furthermore, she was euthyroid. The only pathology associated with the orthotopic thyroid gland was benign palpable nodule and history of thyroid lobectomy for benign goiter. The diagnostic workup was mainly radiographic and immunological in our case.

The prevalence of ectopic thyroid tissue ranges from 7% to 10% [10]. It comprises approximately 1% of mediastinal tumors [11]. Most patients with ectopic thyroid are euthyroid and asymptomatic. However, obstructive or compression-related symptoms, especially of the upper aerodigestive tract, can appear, as can hypothyroidism [12].

Thyroid scintigraphy plays the most important role in diagnosing ectopy, but ultrasonography contributes as well [1]. Surgical excision is the mainstay of the treatment as these tumors usually give rise to compressive symptoms. Thoracotomy or sternotomy is usually required for mediastinal thyroid tumors. Thoracoscopic excision has also been reported [13].

A recent case study from 2007 reported a similar case to current study. They performed lateral thoracotomy to approach the mediastinal lesion. In our patient the tumor had invaded the cortex of the overlying bone, which made it imperative for us to perform a sternal resection [2]. The diagnosis before surgical excision is sometimes difficult in mediastinal masses. Another case reported by shafee S et al had a similar dilemma where they performed the surgery with the initial diagnosis of malignant thymoma, however, the biopsy showed an ectopic papillary carcinoma [14]. It can be attributed to rarity of an ectopic thyroid carcinoma with a normal orthotopic thyroid tissue, hence, warrants the reporting of this case.

## 4 Conclusion

Ectopic thyroid is a rare condition, and its location in the anterior mediastinum is even rarer. The aim of this report is to bring forward malignant ectopic thyroid carcinoma as a differential for malignant mediastinal masses. It is emphasized that the diagnosis is made essentially through physical and pathological examination and, in most cases, only after surgery. A Multidisciplinary approach is recommended in such cases.

## Conflicts of interest

None.

## Funding source

None.

## Ethical approval

The authors were exempted from erc approval by the research council of Aga Khan University Hospital.

## Consent

Written informed consent was obtained from the patient for publication of this case report and accompanying images. A copy of the written consent will be made available for review by the Editor-in-Chief of this journal on request".

## Author contribution

Farida Karim – Study Concept, Data Analysis, Proof Reading.

Usama Khalid Choudry – Critical Review of Literature, Data Interpretation, Article Drafting.

Hina Inam – Study Concept, Study Design, Data Collection, Manuscript Writing.

Saulat hasnain fatmi – Data Anaylsis, Interpretation, Manuscript Drafting.

## Registration of research studies

Not acquired.

## Guarantor

Dr. Saulat Hasnain Fatmi.

## Provenance and peer review

Not commissioned, externally peer-reviewed.
